# The mediation path of physical multimorbidity on the vulnerability to health-related poverty of rural aging families in Ningxia, China: A cross-sectional survey

**DOI:** 10.3389/fpubh.2022.993977

**Published:** 2022-10-18

**Authors:** Wenqin Guo, Jiancai Du, Kexin Chen, Wenlong Wang, Baokai Gao, Zhaoyan Hu, Hui Qiao

**Affiliations:** ^1^School of Public Health and Management, Ningxia Medical University, Yinchuan, China; ^2^Key Laboratory of Environmental Factors and Chronic Disease Control, Yinchuan, China

**Keywords:** physical multimorbidity, poverty vulnerability, propensity score matching, intermediary effect, cross-sectional study

## Abstract

**Background:**

Vulnerability to health-related poverty can predict the probability of families falling into poverty due to health risk impact. In this study, we measured the vulnerability to health-related poverty and examined the mediation path of physical multimorbidity on the vulnerability to health-related poverty of rural aging families in Ningxia, China.

**Methods:**

This cross-sectional study was conducted in Ningxia, China, in February 2019. A multi-stage stratified cluster-randomized design was used to obtain a representative sample in each county. We included participants aged 60 years and older, who had lived there for more than 1 year. A total of 3,653 rural residents older than 60 years old were selected as the research subjects. The three-stage generalized least square method was used to calculate the expected vulnerability to poverty. We used mediating effect model to test the mediation path of poverty vulnerability related to the physical multimorbidity.

**Results:**

Under different poverty line standards, i.e., $1.9/day as low vs. $3.1/day as the high poverty line, the proportion of families that could fall into poverty in the future was 5.3 and 53.7%, respectively. The prevalence of chronic diseases and physical multimorbidity among rural residents >60 years old was 64.62 and 21.24%, respectively. The results of mediating effect test showed that self-rated health status (indirect effect a × b = −0.0052), non-agricultural employment (a × b= −0.0046), household cattle production (a × b = 0.0004), housing type (a × b = −0.0008), gift expenses (a × b = 0.0006) and loan for illness (a × b = 0.0034) were the mediation paths of poverty vulnerability related to the physical multimorbidity.

**Conclusions:**

Concerted efforts are needed to reduce poverty vulnerability related to the physical multimorbidity. The strategy of alleviating poverty should emphasis on promoting non-agricultural employment of vulnerable groups sustainability and developing rural economy, which are important paths to reduce family's vulnerability to health-related poverty.

## Background

In 2013, China proposed the concept of “targeted poverty alleviation,” where poverty caused by health problems was the primary factor (1). The state launched a series of Health Poverty Alleviation Policies, including signing management services for chronic diseases, paying attention to chronic disease prevention and control, and similar (1). It has been found that health-related poverty alleviation had an important role in overall poverty reduction, as in 2020, China completely eliminated absolute poverty (2). However, the problem of relative poverty still remains.

It has been estimated that nearly five million people might be at risk of returning to poverty during the 14th Five Year Plan period (3). The strategic plan for Rural Revitalization also proposes to further alleviate relative poverty by 2035 (4), as unstable poverty relief households and Marginal Poverty households could easily return to poverty again. In fact, different strategies are needed to safeguard this vulnerable groups (5). Focusing on safeguarding relatively poor people from encountering illness-induced poverty are of great significance for consolidation of the achievements of healthy poverty alleviation (6). In July 2017, Ningxia began to promote the alleviation of health-related poverty and implemented a comprehensive health poverty alleviation security policy for patients who become impoverished due to illness and who returned to poverty due to illness (7). According to available data, poverty induced by health problems accounted for 42% nationwide (8). A cluster sampling survey conducted in poor villages in Ningxia showed that 41.5% of the poverty cases were caused by diseases, which become the primary factor for rural families to return to poverty (9). In Guyuan, Ningxia, the patients with chronic diseases accounted for 60.59% of the total number of patients (10).

The economic burden of chronic diseases is an important part of health-related poverty (11). According to the World Health Organization, about 33% of the total disease burden among the elderly aged ≥60 years old in China is attributed to chronic diseases (12). The increasing prevalence of chronic diseases in the elderly and the decline of their ability to work do not only reduce the health capital and labor participation rate (13), but also significantly increase the medical expenditure of aging families (14). It has been estimated that by 2050, the population aging level will reach 30%, the elderly population will exceed 400 million, and the prevalence of chronic diseases among the elderly ≥60 years over in China will reach 75.8% (15). *Physical multimorbidity (suffering from two or more chronic diseases at the same time)* causes high economic costs to individuals and families, and more than one-third of the elderly are chronically illed (16, 17). Suffering from a variety of chronic diseases is significantly related to the increase of catastrophic medical expenditure (18). The high prevalence of chronic diseases and the burden of chronic diseases among the elderly have increased the probability of aging families returning to poverty due to illness, which has become the focus of a series of social policies such as “Healthy China” and “Population aging” (19). Vulnerability to health-related poverty is a prediction of the probability that families might encounter poverty in the future due to health-related issues. These individuals and groups usually share certain social and economic factors that increase their vulnerability to poverty. The vulnerability to health-related poverty can be used as a risk factor or early warning signal of returning to poverty due to illness.

According to previous studies, people living in Western rural areas of China, aging families, those with chronic diseases, and especially people prone to chronic diseases are at high risk of health-related poverty (20–23). The existing literature mainly explored the influencing factors of vulnerability to health-related poverty from the perspective of unexpected health risk (24–27), family resource endowments (28–34), risk response strategies (35–38), and health support system (39). Family resource endowment is the capital on which families depend for survival, including human capital, material capital, and social capital (40). Human capital is divided into education, health, and professional human capital. Previous studies have found that the role of educational human capital in reducing poverty vulnerability is the largest among human capital, which is of great significance for the long-term development of rural residents (4–49). Physical capital can also affect the impact of health risks. The occurrence of chronic diseases is often closely related to public health infrastructure (32). Previous studies have found that public health infrastructure such as clean drinking water and flushing toilets can improve residents' health to a certain extent (50–52).

At present, studies about mediation path of poverty vulnerability related to physical multimorbidity are limited. It is necessary to study the intensity and mode of mediation path. In this study, we measured the vulnerability to health-related poverty among aging families living in rural Western China, examined the net effect of physical multimorbidity on health-related poverty among aging families, and mediation path of vulnerability to poverty related to physical multimorbidity.

## Methods

### Data sources

A total of 5,643 rural residents from 171 villages in four counties of Ningxia, Western China, were surveyed in 2019. The investigation method involved multistage stratified random sampling. All administrative villages in each township of the four sample counties were divided into three levels according to the level of economic development, i.e., high, medium, and low. By using the random number table method, 40% of villages were selected as the sample villages, and 33 rural residents were systematically sampled as the survey samples. The survey method was a face-to-face inquiry survey. The survey subjects were all family members of the sample households. The subjects were rural residents aged >60 years old. People who met the following conditions were selected from the database for inclusion in the study: (1) permanent rural residents who have lived for more than 1 year in the area; (2) elderly ≥60 years old. Finally, 3,653 rural elderly were included in the study. Physical multimorbidity refers to the population with two or more chronic diseases previously diagnosed by doctors. The sample size calculation formula of counting data in descriptive research is $\text{n}=\frac{u_{\alpha }^{2}\pi \left(1-\pi \right)}{\delta^{2}}$, The significance test level α = 0.05 is usually adopted, and the allowable error δ = 0.1π is general. The prevalence of chronic diseases among the elderly in China in 2018 was 59.1% (53), means π = 59.1%, The required sample size is calculated to be 267, The subjects included in this study meet the requirements of sample size.

### Model and variables

#### Explained variable (Y): The vulnerability to health-related poverty

Vulnerability to health-related poverty predicts the probability that families will fall into poverty in the future due to unexpected health issues. The most common measurement method is expected poverty vulnerability (VEP) (41), which mainly uses three-stage feasible generalized least squares (FGLS) to quantify the family's vulnerability to health-related poverty in three following steps:

First, Ordinary Least Square (OLS) is used to estimate the income equation:


(1) $$\begin{array}{l} \ln\;Y_{it+1}=\beta X_{it}+e_{it} \end{array}$$

where

*Y*_*it*+1_ refers to the income level of the rural population in the T+1 period, *X*_*M*_ refers to a series of observable variables that affect the family income level, including family demographic characteristics, health risk variables, family resource endowment variables, risk response strategies, and health support system variables. Considering the heterogeneity of rural population in different counties, townships, and villages, the residual square is regarded as the approximate value of income variance ${\hat{e}}_{\text{i}}^{2}$, and the residual square is used as the explained variable to construct the regression model of residual square ${\hat{e}}_{\text{i}}^{2}$ to individual characteristics:


(2) $${\hat{e}}_{\text{i}}^{2}=\theta \times X_{i}+\eta_{i}$$

The estimated value and residual estimated value of *Y*_*it*+1_ can be obtained through formulas (1) and (2).

Second, heteroscedasticity structure is constructed as a weight for weighted regression, and the expected value (3) and variance (4) of future income logarithm are estimated:


(3) $$\hat{E}\left[\ln Y_{i}|X_{i}\right]=X_{i}\overset{⌢}{\beta }$$

(4) $$\hat{V}\left[\ln Y_{\text{i}}|X_{\text{i}}\right]={\overset{⌢}{\sigma }}_{ei}^{2}=X_{i}\overset{⌢}{\theta }$$

Finally, the poverty line is selected to estimate the vulnerability to poverty. This study used the international poverty line of $1.9/day and $3.1/day (34) as the poverty lines for measuring the vulnerability to poverty. The value of health poverty vulnerability was distributed between zero and one. Those who scored ≥0.5 were categorized as families highly vulnerable to poverty and those <0.5 as families with vulnerability to poverty (42, 43). The research subjects were aging rural families, so the lognormal distribution was more applicable. The logarithm of the poverty lineIn*l*is in formula (5):


(5) $${\overset{⌢}{v}}_{i}=\overset{⌢}{P}\left({\text{lnY}}_{\text{i}}<\ln l|X_{\text{i}}\right)=\varphi \left(\frac{\ln l-X_{\text{i}}\hat{\beta }}{\sqrt{X_{\text{i}}\hat{\theta }}}\right)$$

Tobit model identifies health poverty vulnerability risk factors:

The value of vulnerability to health-related poverty is a limited continuous dependent variable. Therefore, the Tobit model was selected to screen the significant influencing factors of vulnerability to poverty. The measured value of vulnerability to poverty was taken as the dependent variable (V), and family demographic characteristics, health risk impact, family resource endowment, family risk response strategy and health support system are taken as the explanatory variable (X_j_).


(6) $$\begin{array}{l} V=\beta 0+\beta jXj+\varepsilon \end{array}$$

#### Explanatory variable (X): Physical multimorbidity

Physical multimorbidity *(suffering from two or more chronic diseases at the same time)*, The indicators of physical multimorbidity were obtained through the following questions in the questionnaire: “did you have a chronic disease diagnosed by a doctor in the past?”, “if so, what are the diseases, and fill in the names of the three most serious diseases.” We counted the number of chronic diseases in each participant to identify people who with physical multimorbidity.

Taking the elderly with physical multimorbidity as the treatment group and the elderly without physical multimorbidity as the control group, the propensity score matching method was used to match the two groups of *Control Variables* in order to maximize the control of confounding factors and data bias. First, the propensity scores of the treatment group and the control group were estimated. The logit model was used to calculate the tendency score, as follows:


(7) $$\begin{array}{l} L\text{o}git\left(\text{Multimorbidity}\right)=\beta_{0}+\beta_{1}X_{h}+\varepsilon_{h} \end{array}$$

Second, a balance test and common support test were performed. A balance test was used to test whether there was a significant difference in each covariate between the matched treatment group and the control group and whether there was a significant difference in the joint distribution of covariates before and after matching. A common support test was used to ensure that propensity scores overlapped more between the treatment group and the control group (44). Finally, the “average treatment on the treated (ATT)” was obtained, i.e., the net effect of physical multimorbidity on poverty vulnerability.

#### Mediating variable (M): Mechanism test of mediating effect model

The family's vulnerability to health-related poverty was used as the explanatory variable (Y), *Physical multimorbidity* as the explanatory variable (X), and human capital (Education level, The average length of education of family, Self-rating health, Non-agricultural workers), material capital (Household livestock ownership, Housing type, Type of drinking water, Toilet type, Separation of housing and kitchen) and social capital (Gift expenses) in resource endowment as the intermediary variable (M_1_) to test the possible path of family resource endowment in alleviating the impact of health risk on family's vulnerability to poverty. Taking the low insured households and medical assistance of risk response strategy as intermediary variables (M_2_), this study tested the role path of risk response strategy in alleviating health impact. Taking the transit time and chronic disease diagnosis and treatment institutions in the health support system as intermediary variables (M_3_), we tested their role in alleviating the health impact (Table 1).

**Table 1:** Variable definition.

**Variables**		**Explanation**	**Code**
Explained variable (Y)		Health poverty vulnerability	Measure with expected poverty vulnerability (VEP)
Explanatory variable (X)		Physical multimorbidity	1 = treatment group, 2 = control group
Control variable (C)		Gender	1 = male, 2 = female
		Age	continuous variable
		Marital status	1 = unmarried, 2 = married, 3 = divorced, 4 = widowed, 5 = other
		Family size	Household population
		Number of the labor force	The family working-age population, 15–64 years old
		Dependency ratio	1–labor force / family size
Resource endowment (M_1_)	Human capital	Education level	1 = no schooling, 2 = primary school, 3 = junior high school, 4 = senior high school or above
		The average length of education of family(year)	No schooling = 0, primary school = 6, junior middle school = 9, senior high school and above = 12
		Self-rating health	1 = very good, 2 = good, 3 = average, 4 = poor, 5 = very poor
		Non-agricultural workers	1 = yes, 2 = no
	Material capital	Household livestock ownership (cattle)	continuous variable
		Housing type	1 = brick soil concrete, 2 = brick wood, 3 = Civil Engineering, 4 = full brick, 5 = cave
		Type of drinking water	1 = tap water, 2 = mountain spring water, 3 = hand press well water, 4 = cellar water, 5 = well water, 6 = River and lake water, 7 = pond and ditch water
		Toilet type	1 = water flushing type, 2 = biogas, 3 = Double urn funnel type, 4 = deep pit, 5 = toilet, 6 = dry toilet, 7 = no toilet
		Separation of housing and kitchen	1 = yes, 2 = no
	Social capital	Gift expenses(log)	Continuous variable (logarithm)
Risk response strategy (M_2_)	Informal	Income from migrant workers	Continuous variable (logarithm)
		loans because of illness	1 = yes, 2 = no
	Regular	Low-income households	1 = yes, 2 = no
		Medical assistance	1 = yes, 2 = no
Health support system (M_3_)	Health service accessibility	Physical accessibility	1≤30 min, 2 = 30–60 min, 3 = 60–90 min, 4 = >90 min
	Availability of health services	Chronic disease diagnosis and treatment institutions	1 = village clinics, 2 = township hospitals, 3 = county hospitals, 4 = private clinics, 5 = others

The intermediary effect model can analyze the process and mechanism of the influence between variables. When studying the influence of explanatory variable X *(Physical multimorbidity)* on the explained variable Y *(The vulnerability to health-related poverty), Physical multimorbidity* not only has a direct impact on *the vulnerability to health-related poverty*, but also an indirect impact on *the vulnerability to health-related poverty* through variable M (*Mediating variable)*; thus, M can be called the intermediary variable, and the model *X* → *M* → *Y* reflecting the relationship between the three is called the intermediary effect model (45, 46). The mediating effect can be expressed as the product of coefficient b and coefficient a × b. This product term indicates how much of the effect of X on Y reaches Y through M. The independent variable of this study was category variable, so we used regression analysis to conduct intermediary analysis according to the stepwise method (54).


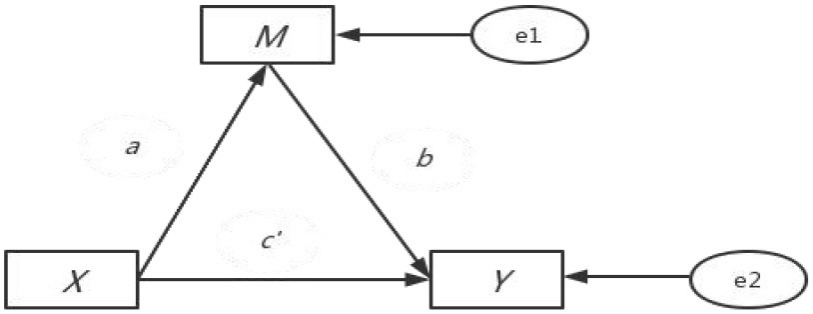



$$\begin{array}{l} M=aX+e_{1}\;Y={\text{c}}^{\prime }X+bM+e_{2} \end{array}$$

#### Control variable (C)

Gender, age, marital status, family size, labor force, and dependency ratio were used as control variables in family demographic characteristics (Table 1).

## Results

### Description of vulnerability to poverty among aging rural families

**Figure 1:**
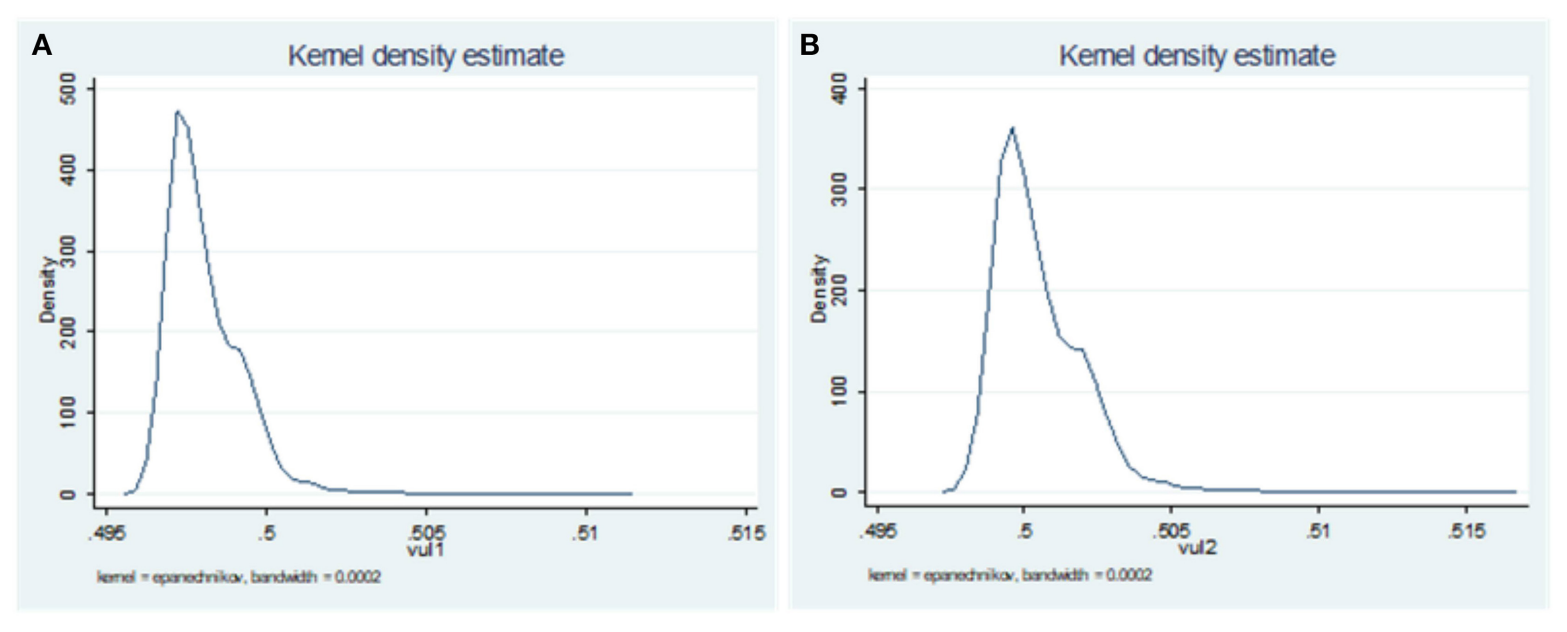
Kernel Density of vulnerability to poverty. **(A)** VEP_1_-poverty line = 1.9$; **(B)** VEP_2_-poverty line = 3.1$.

The vulnerability to poverty of aging rural elderly families calculated with the international poverty line of $1.9/day was 0.498 ± 0.001, and the proportion of vulnerable (VEP_1_) families was 5.3%. The vulnerability to poverty value of aging rural families calculated with the international poverty line of $3.1/day was 0.5 ± 0.001, and the proportion of vulnerable (VEP_2_) families was 53.7% (Table 2; Figure 1). Taking *Poverty vulnerability value (3.1$)* as the explanatory variable, family size, the number of the family labor force, dependency ratio, the toilet type in material capital and housing are separated from the kitchen, the logarithm of gift expenditure in social capital, the income of migrant workers in risk response strategy, loans due to illness, low-income households, the level of chronic disease diagnosis and treatment institutions providing medical assistance and health support system all resulted as the factors affecting vulnerability to poverty. The level of chronic disease diagnosis and treatment institutions providing the medical assistance and health support system resulted as factors affecting vulnerability to poverty (Table 3).

**Table 2:** Description of basic family situation and vulnerability to poverty.

**Variable**	**Observation**	**Mean**	**Std. Dev**.	**Min**	**Max**
Poverty vulnerability value_1_ ($\bar{X}\pm S$)	3,653	0.498	0.001	0.496	0.511
Poverty vulnerability value_2_ ($\bar{X}\pm S$)	3,653	0.500	0.001	0.497	0.516
VEP_1_* (%)	3,653	0.053	0.225	0	1
VEP_2_ (%)	3,653	0.537	0.499	0	1
poverty line_1_ (1.9$) (ln)	3,653	8.390	0.000	8.390	8.390
poverty line_2_ (3.1$) (ln)	3,653	8.880	0.000	8.880	8.880

* VEP_1_, the proportion of expected vulnerability to poverty calculated with the international poverty line of $1.9/day.

VEP_2_, the proportion of expected vulnerability to poverty calculated with the international poverty line of $3.1/day.

**Table 3:** Analysis on influencing factors of poverty vulnerability (3.1$).

***Poverty vulnerability value (3.1$)***	**OR**	**St. Err**.	***t*-value**	***p*-value**	**[95% Conf]**	**Interval**	**Sig**
Gender	−0.009	0.007	−1.29	0.196	−0.022	0.005	
Age	0.000	0.001	−0.60	0.552	−0.001	0.001	
Marital status	0.003	0.005	0.57	0.571	−0.006	0.012	
Family size	0.067	0.004	17.42	< 0.001	0.059	0.075	***
Number of labor force	−0.049	0.006	−7.76	< 0.001	−0.061	−0.036	***
Dependency ratio	−0.147	0.019	−7.89	<0.001	−0.183	−0.11	***
Education level	0.001	0.006	0.16	0.870	−0.011	0.013	
Average length of education of family	−0.002	0.002	−1.43	0.152	−0.006	0.001	
Self–rating health	−0.001	0.004	−0.29	0.775	−0.008	0.006	
Non–agricultural workers	0.016	0.014	1.15	0.249	−0.011	0.044	
Household livestock ownership (cattle)	−0.001	0.001	−1.23	0.220	−0.004	0.001	
Housing type	0.003	0.003	1.04	0.300	−0.002	0.008	
Type of drinking water	0.004	0.003	1.43	0.151	−0.001	0.009	
Toilet type	0.010	0.003	3.61	<0.001	0.004	0.015	***
Separation of housing and kitchen	0.014	0.007	2.00	0.046	0.000	0.028	**
Gift expenses (log)	−0.032	0.001	−31.25	<0.001	−0.034	−0.03	***
Income from migrant workers	−0.003	0.001	−3.48	0.001	−0.004	−0.001	***
Loans because of illness	0.029	0.008	3.74	<0.001	0.014	0.044	***
Low–income households	0.018	0.007	2.81	0.005	0.006	0.031	***
Medical assistance	0.029	0.011	2.72	0.007	0.008	0.050	***
Physical accessibility	0.002	0.003	0.56	0.579	−0.005	0.008	
Chronic disease diagnosis and treatment institutions	−0.011	0.006	−1.88	0.060	−0.022	0.000	*
Constant	0.005	0.061	0.08	0.938	−0.114	0.123	

*** *p* < 0.01;

** *p* < 0.05;

* *p* < 0.1.

### Analysis of tendency score matching results

**Figure 2:**
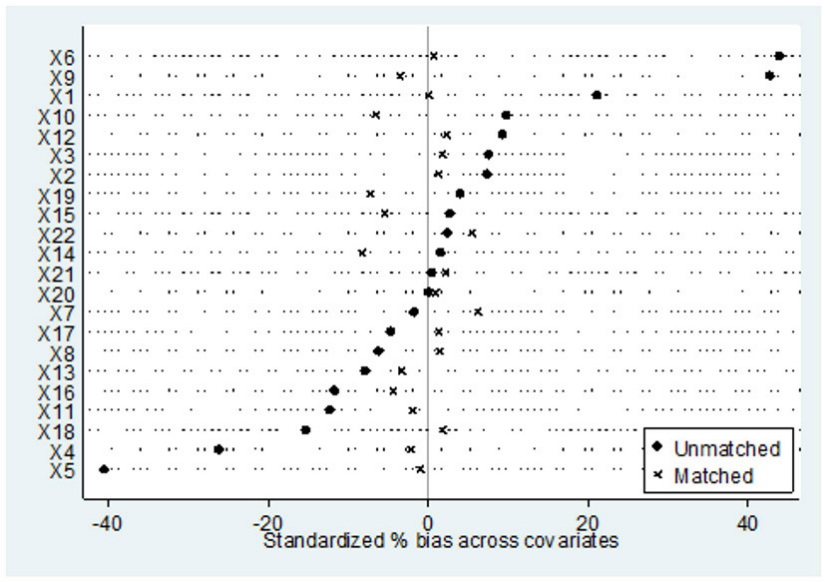
Standardized deviation diagram of covariates.

**Figure 3:**
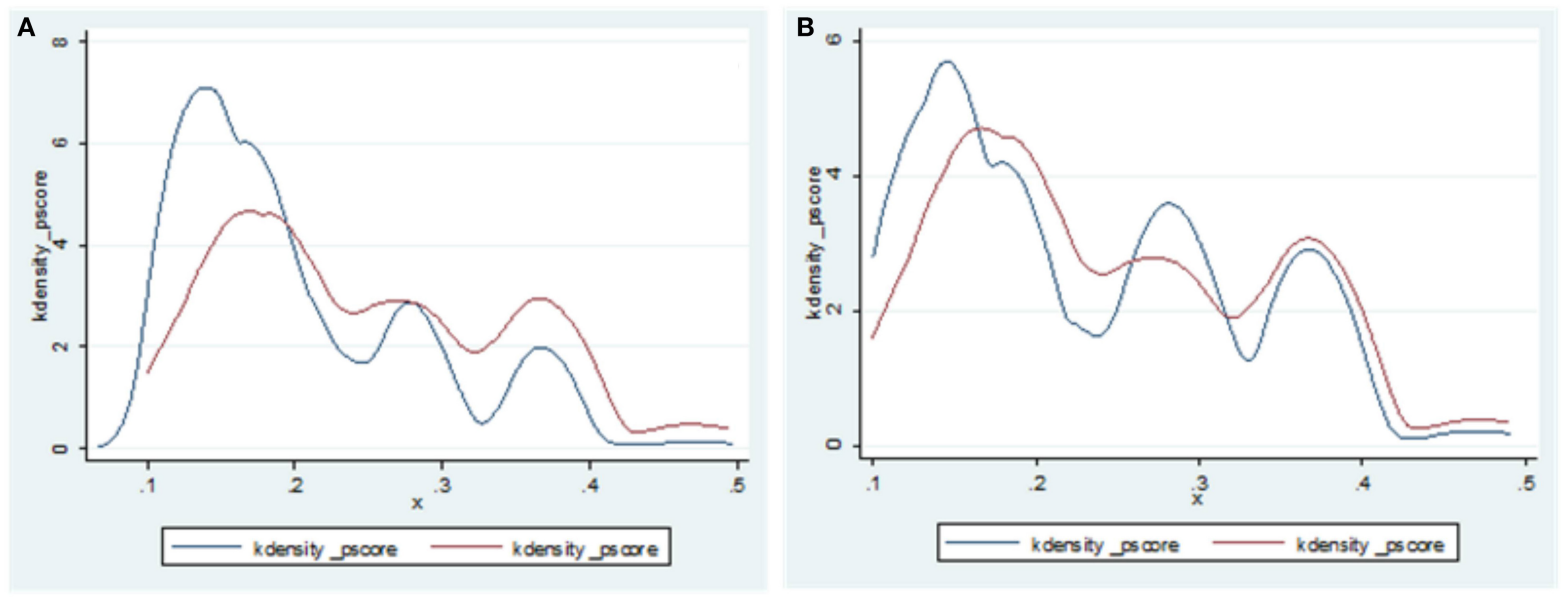
Balance test before **(A)** and after **(B)** matching between treatment group and control group.

**Figure 4:**
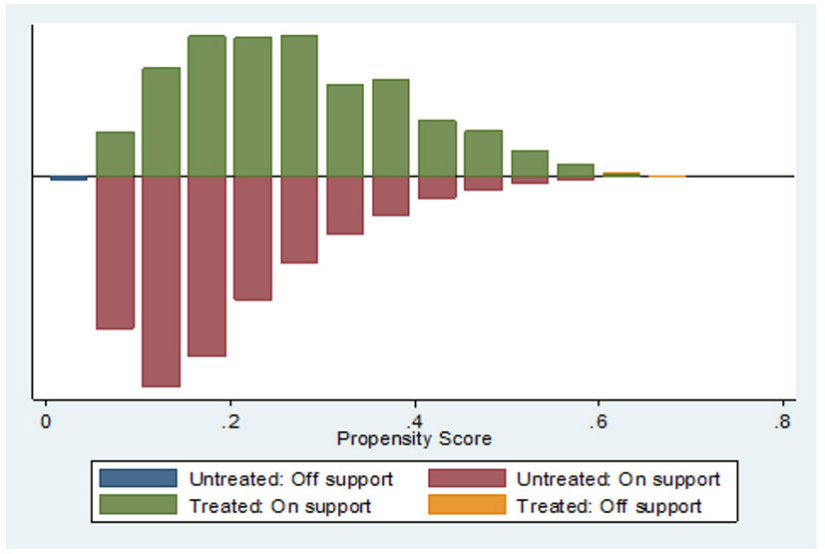
Common support test after matching between treatment group and control group. Untreated off support = 15; Treated off support = 2.

The prevalence of chronic diseases among rural elderly people > 60 years old was 64.62%, and the prevalence of physical multimorbidity was 21.24%. Nearest neighbor matching (1:1 ratio) was used to calculate the average treatment effect (ATT) of the treatment group. Before calculating ATT, a balance test and common support test were performed. As shown in Figures 2, 3 depicting the balance test results, the covariate standardization deviation (% bias) of the matched post-processing group and the control group was greatly reduced (both <10%). Figure 4 shows the results of the common support test, where the two groups of samples were basically in the common support range, while 15 samples in the control group were not in the common support range vs. only two samples in the treatment group. As shown, most of the observed values were in the common value range (on support), and the tendency score had greater overlap in the processing group and the control group.

As shown in Table 4, for the vulnerability to poverty of aging rural families calculated at the international poverty line of $1.9/day, the vulnerability to poverty of the matched post-treatment group and the control group were 0.043 and 0.032, respectively. The difference was 0.011, i.e., the net effect of the coexistence of chronic diseases on the vulnerability to poverty of aging rural families was 0.011. According to the vulnerability to poverty value of rural aging families calculated at the international poverty line of $3.1/day, the vulnerability to poverty values of the matched post-treatment group and the control group were 0.486 and 0.474, respectively. The difference was 0.012, i.e., the net effect of physical multimorbidity on the vulnerability to poverty of rural elderly families was 0.012. The results show that the difference of vulnerability value between the treatment group and the control group was 0.012, that is, compared with the families without chronic diseases and with only one chronic disease, the poverty vulnerability value of families with physical multimorbidity will increase by 0.012. This shows that families with physical multimorbidity will increase the probability of poverty vulnerability.

**Table 4:** The net effect of physical multimorbidity on poverty vulnerability [neighbor (K = 1)].

	**Before and after matching**	**Treated**	**Controls**	**Difference**	**S.E**.	**T–stat**
VEP_1_ (poverty line = 1.9$)	Unmatched	0.043	0.056	−0.014	0.009	−1.520
	ATT	0.043	0.032	0.011	0.018	0.570
VEP_2_ (poverty line = 3.1$)	Unmatched	0.486	0.551	−0.065	0.020	−3.210
	ATT	0.486	0.474	0.012	0.042	0.280

### Analysis of intermediary effect of action path

Based on the analysis of the net effect of physical multimorbidity on poverty vulnerability, it is necessary to examine the mediation path further. As shown in Table 5, In the path with human capital as intermediary variable, the indirect effect of average level of family education was 0.0003, the contribution rate was 41.75%; self-rating health was −0.0052, the contribution rate was 82.80%; non-agricultural employment was −0.0046, the contribution rate was 2.57%. In the path with material capital as intermediary variable, the indirect effect of household cattle production was 0.0004, the contribution rate was 1.95%; Housing type was −0.0008, the contribution rate was 4.81%. In the path with social capital as intermediary variable, the indirect effect of gift expenses was 0.0006, the contribution rate was 0.8%; In the path with Risk Response Strategy as intermediary variable, the indirect effect of loans because of illness is 0.0034, the contribution rate was 2.17%. Analysis shows that self-rated health status, non-agricultural employment, household cattle production, housing type, gift expenses and loan for illness were the mediation paths of poverty vulnerability related to physical multimorbidity. These paths had a partial mediating role in the process of health-related vulnerability to poverty. Among these paths, the mediating effect of human health capital in family resource endowment was the largest, accounting for 82.8%; however, the total effect C was not significant. The sign of indirect effect a × b was opposite to that of direct effect C', indicating that the mediating effect of self-rated health results in vulnerability to poverty related to the physical multimorbidity existing in masking effect. The intermediary effect, direct effect, and total utility of non-agricultural employees in professional human capital in family resource endowment were statistically significant. Non-agricultural employment reduced the incidence of vulnerability to poverty. Household livestock ownership and housing type in physical capital, gift expenditure in social capital and disease-related lending in risk response strategy also had a part in the intermediary effect. Disease-related lending alleviated the vulnerability to health-related poverty.

**Table 5:** Intermediary effect test of vulnerability to poverty related to the physical multimorbidity.

**VEP_2_**	**a coefficient**	**b coefficient**	Indirect effect (a*b)	**Direct effect**	**Total effect**	**The proportion of total effect that is mediated**
Education level	0.0041	0.0658	0.0003	−0.0808***	−0.0805***	−0.00335
Average length of education of family	0.0038	0.0647***	0.0003	0.0003	0.0006	0.4175
Self–rating health	−0.0751***	0.0691***	−0.0052***	0.0115	0.0063	−0.8280
Non–agricultural workers	−0.0677**	0.0687***	−0.0046*	0.1851***	0.1805***	−0.0257
Household livestock ownership (cattle)	0.006902 ***	0.0580***	0.0004**	0.0201 ***	0.0205***	0.0195
Housing type	−0.0130**	0.0629***	−0.0008*	−0.0162**	−0.0170**	0.0481
Type of drinking water	0.0099*	0.0622***	0.0006	0.0255***	0.0262***	0.0236
Toilet type	−0.0021	0.0659***	−0.0001	0.0659***	0.0658***	−0.0021
Separation of housing and kitchen	−0.0096	0.0666***	−0.0006	0.1451***	0.1444***	−0.0044
Gift expenses (log)	0 0.0060***	0.0935***	0.0006***	−0.0711***	−0.0705***	−0.0080
Income from migrant workers	0.0019	0.0643***	0.0001	0.0024	0.0025	0.0482
loans because of illness	0.0617***	0.0547***	0.0034**	0.1518***	0.1552***	0.0217
Low–income households	−0.0134	0.0672***	−0.0009	0.1228***	0.1219***	−0.0074
Medical assistance	0.0001	0.0647***	0.000008	0.0476*	0.0476*	0.0002
Physical accessibility	−0.0007	0.0649***	−0.000043	0.0487***	0.0487***	−0.0009
Chronic disease diagnosis and treatment institutions	−0.0081	0.0636***	−0.0005	−0.0764***	−0.0770***	0.0067

*** *p* < 0.01;

** *p* < 0.05;

* *p* < 0.1.

## Discussion

We measured the vulnerability to health-related poverty of aging rural families using two poverty lines and analyzed the mediation path of physical multimorbidity on poverty vulnerability based on the survey data obtained in 2019 from aging rural families in Ningxia, China. The empirical analysis showed that: first, Taking the high poverty line as the standard, more than half of the households were vulnerable; second, Families with physical multimorbidity were more vulnerable than those without chronic diseases or with one chronic disease; third, the test of intermediary effect mechanism revealed that self-rated health status, non-agricultural employment, household cattle production, housing type, gift expenses and loan for illness were important ways to reduce family's vulnerability to health-related poverty.

Taking the vulnerability to poverty measured by the high poverty line as the explanatory variable, households with large population had a higher probability of poverty vulnerability; Households with large household labor force are less vulnerable. The type of toilet and the separation of kitchen and housing were risk factors of poverty vulnerability. Previous studies have found that labor migration can significantly reduce the family's vulnerability to health-related poverty (55, 56). Our study also revealed that income of migrant workers was associated with the vulnerability to poverty of family. It has been reported that low-income households and medical assistance have no impact on the vulnerability to poverty (57), which was contrary to our results that low-income households and medical assistance could reduce vulnerability to health-related poverty. We also found that the level of chronic disease diagnosis and treatment institutions were risk factors of the vulnerability to poverty. Suppose grass-roots medical institutions cannot meet the medical needs of chronic patients, thus making a considerable number of patients seek medical help from institutions above the county level. In that case, this tends to increase the disease economic burden of families of chronic patients, affecting their vulnerability to health-related poverty (1).

Intermediary effect mechanism test revealed that family human capital, material capital, social capital, and private lending in risk response strategy were important ways to reduce family's poverty vulnerability related to physical multimorbidity. It has been found that the deterioration of residents' health levels makes them face higher vulnerability to poverty. For every 10% decline in residents' health level, the vulnerability to poverty increases by 6% (58). Some previous studies have also found that self-rated health status was associated with the vulnerability to health-related poverty (59), which is consistent with the results of the present study. Engaging in non-agricultural work and social capital can help to reduce farmers' the vulnerability to poverty. Individual differences in non-agricultural employment and health contribute the most to vulnerability to poverty (60). Our results revealed that non-agricultural employees was an important intermediary path to reduce the incidence of the vulnerability to health-related poverty. Material capital can also help to cope with the impact of health risks. The amount of household livestock and other realizable assets can be used to measure material capital, which reflects the economic situation of families to a certain extent and may alleviate effect on the vulnerability to health-related poverty (28). Gift spending in social capital also participates in the intermediary effect. Existing studies have reported that social capital has an external driving force on the family's livelihood capital (61). In the risk response strategy, lending due to illness also has a part of the intermediary effect, and it is an intermediary path to alleviates the vulnerability to health-related poverty. Some scholars have found that the intermediary effect of private lending is about 10%, which can reduce the vulnerability to poverty (36).

### Implications for policy and practice

Understanding the intermediary path of poverty vulnerability related to physical multimorbidity of aging rural families may help to reduce the risk of vulnerable groups returning to poverty: first, To prevent the further development of chronic diseases and block the path of poverty, risk factors of multimorbidity such as dietary control, abstaining from tobacco, alcohol and physical activities should be targeted at an early state to prevent or delay the disease onset (62). The resource endowment of families is largely affected by the subjective initiative of family members through hard work, and non-agricultural employment, as improving labor skills can greatly enhance the family's ability to generate income (1), promote the development of rural labor economy and rural community construction, and form a good economic and social environment to improve farmers' ability to resist risks. Second, strategies for alleviation of systematic poverty should focus on promoting non-agricultural employment among vulnerable groups and continue to block the poverty trap through poverty alleviation in education and health. It is also necessary to focus on constructing rural family human capital. The higher the education level of rural residents, the lower their vulnerability to poverty (63). Education input-output is a relatively long-term process, and there is a lag effect on the embodiment of poverty alleviation effect. Third, the governance scheme for the health poverty vulnerability of rural elderly families should focus on improving the family coping ability. Primary care facilities should be strengthened to increase availability and accessibility while making the facilities affordable (64).

### Strengths and limitations

Although we made a preliminary discussion on mediation path of the physical multimorbidity on vulnerability to poverty, due to the limited availability of data, the robustness of the conclusions of empirical research need to be further improved. With the emergence of higher quality data, the measurement of vulnerability to poverty or residents' vulnerability to health-related poverty and the discussion of relevant internal mechanisms could be further improved.

## Conclusions

In the study, the health-related poverty vulnerability index was introduced as the risk warning signal of returning to poverty due to illness. In the process of poverty vulnerability related to the physical multimorbidity, self-rated health status, non-agricultural employment, household cattle production, housing type, gift expenses and lending due to illness were intermediary paths to alleviate vulnerability to poverty.

## Data availability statement

The original contributions presented in the study are included in the article/supplementary material, further inquiries can be directed to the corresponding author/s.

## Ethics statement

Ethical approval was granted by the Ethics Committee of Ningxia Medical University, Approval number, No. 2018-114. The patients/participants provided their written informed consent to participate in this study.

## Author contributions

HQ conceptualized the research idea and design. WG participated in the research design, drafted the manuscript, analyzed, and interpreted the data. JD helped revise the manuscript and interpreted the data. KC, WW, BG, and ZH helped clean the data. All authors contributed to the article and approved the submitted version.

## Funding

This paper was supported by the National Natural Science Foundation of China (No. 71864030).

## Conflict of interest

The authors declare that the research was conducted in the absence of any commercial or financial relationships that could be construed as a potential conflict of interest.

## Publisher's note

All claims expressed in this article are solely those of the authors and do not necessarily represent those of their affiliated organizations, or those of the publisher, the editors and the reviewers. Any product that may be evaluated in this article, or claim that may be made by its manufacturer, is not guaranteed or endorsed by the publisher.

## Abbreviations

VEP: expected poverty vulnerability
FGLS: feasible generalized least squares
OLS: Ordinary Least Square
VEP_1_: the expected poverty vulnerability calculated with the international poverty line of $1.9/day
VEP_2_: the proportion of expected poverty vulnerability calculated with the international poverty line of $3.1/day
ATT: average treatment effect.
